# Effects of Secretome from Fat Tissues on Ion Currents of Cardiomyocyte Modulated by Sodium-Glucose Transporter 2 Inhibitor

**DOI:** 10.3390/molecules25163606

**Published:** 2020-08-08

**Authors:** Shih-Jie Jhuo, I-Hsin Liu, Wei-Chung Tsai, Te-Wu Chou, Yi-Hsiung Lin, Bin-Nan Wu, Kun-Tai Lee, Wen-Ter Lai

**Affiliations:** 1 Division of Cardiology, Department of Internal Medicine, Kaohsiung Medical University Hospital, Kaohsiung 80708, Taiwan; jhuoshihjie@gmail.com (S.-J.J.); ihsin343@yahoo.com.tw (I.-H.L.); azygo91@gmail.com (W.-C.T.); tewuchou@gmail.com (T.-W.C.); caminolin@gmail.com (Y.-H.L.); wtlai@kmu.edu.tw (W.-T.L.); 2 Graduate Institute of Clinical Medicine, Kaohsiung Medical University, Kaohsiung 80708, Taiwan; binnan@kmu.edu.tw; 3 Department of Internal Medicine, Faculty of Medicine, College of Medicine, Kaohsiung Medical University, Kaohsiung 80708, Taiwan

**Keywords:** pericardial fat, adipocytokine, delayed-rectifier potassium current, l-type calcium channel current

## Abstract

Sodium-glucose transporter 2 (SGLT2) inhibitors were shown to decrease mortality from cardiovascular diseases in the EMPA-REG trial. However, the effects of empagliflozin (EMPA) for cardiac arrhythmia are not yet clarified. A total of 20 C57BL/6J mice were divided into four groups: (1) The control group were fed standard chow, (2) the metabolic syndrome (MS) group were fed a high-fat diet, (3) the empagliflozin (EMPA) group were fed a high-fat diet and empagliflozin 10 mg/kg daily, and (4) the glibenclamide (GLI) group were fed a high-fat diet and glibenclamide 0.6 mg/kg daily. All mice were sacrificed after 16 weeks of feeding. H9c2 cells were treated with adipocytokines from the pericardial and peripheral fat from the study groups. The delayed-rectifier potassium current (I_K_) and L-type calcium channel current (I_Ca,L_) were measured by the whole-cell patch clamp techniques. Adipocytokines from the peripheral and pericardial fat tissues of mice with MS could decrease the I_K_ and increase the I_Ca,L_ of cardiomyocytes. After treating adipocytokines from pericardial fat, the I_K_ in the EMPA and GLI groups were significantly higher than that in the MS group. The I_K_ of the EMPA group was also significantly higher than the GLI group. The I_Ca,L_ of the EMPA and GLI groups were significantly decreased overload compared with that of the MS group. However, there was no significant difference of I_K_ and I_Ca,L_ among study groups after treating adipocytokines from peripheral fat. Adipocytokines from pericardial fat but not peripheral fat tissues after EMPA therapy attenuated the effects of I_K_ decreasing and I_Ca,L_ increasing in the MS cardiomyocytes, which may contribute to anti-arrhythmic mechanisms of sodium-glucose transporter 2 (SGLT2) inhibitors.

## 1. Introduction

Metabolic syndrome (MS) is a well-known risk factor for cardiovascular diseases and cardiac arrhythmias. The modification of electrophysiological substrates and autonomic nervous function in patients with MS may result in cardiac arrhythmias [1,2]. The relationship of MS and pericardial fat has been extensively discussed in many reports [3,4]. Several studies have demonstrated that the amount of pericardial fat is associated with the occurrence of atrial fibrillation (AF) and that catheter ablation of the pericardial fat pad can decrease the occurrence of AF [5]. Another study also showed that a high-fat diet could increase the risk of ventricular arrhythmias [6]. Adipose tissues were not only considered a deposit of energy but also as an endocrine organ responsible for the secretion of bioactive molecules termed “adipocytokines” [7]. Previous studies have shown that adipocytokines, either from peripheral or pericardial fat, may comprise several cytokines and proinflammatory mediators that could modulate the genesis of atrial and ventricular arrhythmias [5,6]. Mazurek et al. investigated human epicardial adipose tissue and found that epicardial adipose tissue was a source of several cytokines, including monocyte chemotactic protein 1, interleukin (IL)-1β, IL-6, and tumor necrosis factor (TNF)-α, all of which may promote the genesis of atherosclerosis [7]. The adipocytokines and metabolites of pericardial fat also could result in mitochondrial dysfunction, autonomic nervous dysfunction, and cardiomyocyte death, all of which might lead to heart failure [8]. Our previous study also demonstrated that adipocytokines from pericardial fat could affect the ion currents of cardiomyocytes, which might contribute to the mechanisms of arrhythmogenesis [9].

Empagliflozin (EMPA), a sodium-glucose transporter 2 (SGLT2) inhibitor, which inhibits glucose reabsorption in renal tubules, is a new generation of the glucose-lowering agent [10]. Type 2 diabetes mellitus (DM) patients receiving EMPA therapy had a lower incidence of cardiovascular disease and heart failure compared with those patients not receiving EMPA therapy in the EMPA-REG study [10]. In addition to the glucose-lowing effect, SGLT2 inhibitors also reduced the amount of pericardial fat [11], had an antihypertensive effect, and modified autonomic nervous function [12]. By consensus report of the American Diabetes Association and the European Association of the Study of Diabetes published in 2018, SGLT2 inhibitors were suggested to be the first consideration for use in DM patients with cardiovascular disease and heart failure [13]. However, whether SGLT2 inhibitors have antiarrhythmic effects remains uncertain. Previous studies have shown that EMPA did not prolong the QT (from the initiation of Q wave to the end of T wave) interval of electrocardiography and could alleviate atrial remodeling and improve mitochondrial function in diabetic rats [14,15]. Alterations of the ion currents of cardiomyocytes are one of the arrhythmogenic mechanisms. We hypothesized that EMPA could affect the ion currents of cardiomyocytes by modifying the effective strength of adipocytokines on the ion currents of cardiomyocytes. The purpose of this study was to determine the effects of adipocytokines from different adipose tissues of metabolic mice receiving EMPA therapy on the ion currents of cardiomyocytes.

## 2. Results

### 2.1. The Characteristics of Study Groups

The mean body weights at the time of starting feeding (age: 8 weeks) were 22.61 ± 1.17, 24.01 ± 0.30, 24.14 ± 1.06, and 23.42 ± 0.93 g in the control, MS, EMPA, and glibenclamide (GLI) groups, respectively. The body weights were not significantly different among the study groups at the time of starting feeding (*p* = 0.15). Table 1 demonstrates the characteristics of the study groups at the time of collecting adipose tissues. Compared with the control group, the mean body weight was significantly higher in the MS, EMPA, and GLI groups (27.17 ± 1.02 g vs. 42.61 ± 2.27, 38.12 ± 4.50, and 38.88 ± 5.21 g, respectively; all *p* < 0.01). The mean body weight was not significantly different among the MS, EMPA, and GLI groups. The mean fasting glucose level of the GLI group was significantly higher than those of the control, MS, and EMPA groups (180.40 ± 17.70 mg/dL vs. 144.60 ± 21.98 mg/dL; *p* = 0.02, 155.00 ± 11.86 mg/dL; *p* = 0.02, 130.50 ± 20.51 mg/dL; *p* = 0.04, respectively). The mean fasting glucose levels were not significantly different among the control, MS, and EMPA groups. Compared with the control group, the mean total cholesterol, low-density lipoprotein, and high-density lipoprotein levels in the MS and GLI groups but not the EMPA group were significantly higher (all *p* < 0.05, Table 1). The mean triglyceride levels were not significantly different among the control, MS, and EMPA groups. The mean triglyceride level in the GLI group was significantly lower than that in the control group (36.19 ± 8.91 vs. 64.77 ± 5.35 mg/dL; *p* < 0.01). The alterations of the body weight and lipid profiles among the study groups suggested that the MS in mice could be induced after feeding a high-fat diet for 16 weeks. The mean collected fat tissue weights were 0.09 ± 0.01, 0.11 ± 0.02, 0.09 ± 0.02, and 0.14 ± 0.07 g in the control, MS, EMPA, and GLI groups, respectively (*p* = 0.26).

### 2.2. Delayed-Rectifier Potassium Outward Currents (I_K_) and L-Type Calcium Channel Current (I_Ca,L_) in H9c2 Cells

**Figure 1:**
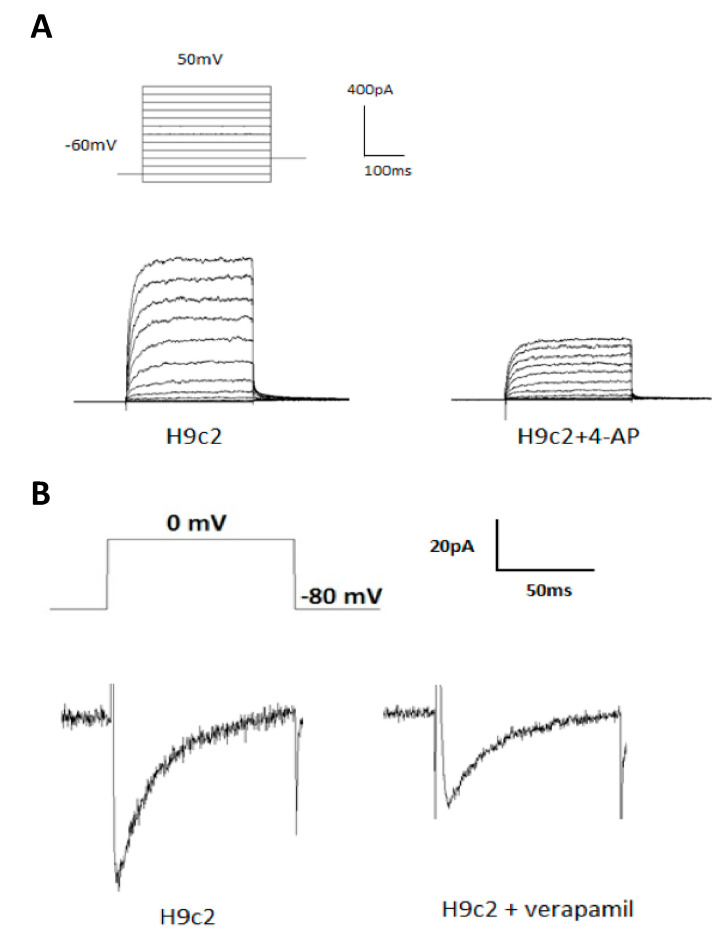
Delayed-rectifier potassium current (I_K_) and L-type calcium channel current (I_Ca,L_) in H9c2 cells. (**A**) Representative recordings of I_K_ elicited by 300 ms of depolarizing step pulses from −70 to 50 mV at a holding potential of −60 mV in H9c2 cells. The I_K_ was significantly suppressed by 4-aminopyridine (4-AP) (5 mM). (**B**) Representative recordings of the I_Ca,L_ evoked by applying a pulse of 0 mV for 300 ms at a holding potential of −80 mV in H9c2 cells. The I_Ca,L_ was suppressed significantly by verapamil (1 µM).

The I_K_ was evoked in H9c2 cells with 300 ms of depolarizing step pulses from −70 to 50 mV with 10 mV increments at a holding potential of −60 mV. The I_K_ first elucidated significantly at the potential of −20 mV and the amplitude of the currents increased with more positive membrane potentials until it reached membrane potentials of 50 mV as described in our previous study [7]. The I_K_ was suppressed significantly by 5 mM 4-aminopyridine (4-AP) (Figure 1A).

As described in the previous study [12], a depolarizing single pulse with 0 mV was applied for 300 ms at a holding potential of −80 mV to measure the I_Ca,L_ in H9c2 cells. The I_Ca,L_ was elucidated and significantly suppressed by verapamil (1 µM) (Figure 1B).

### 2.3. Effects of Adipocytokines on I_K_ in H9c2 Cells

**Figure 2:**
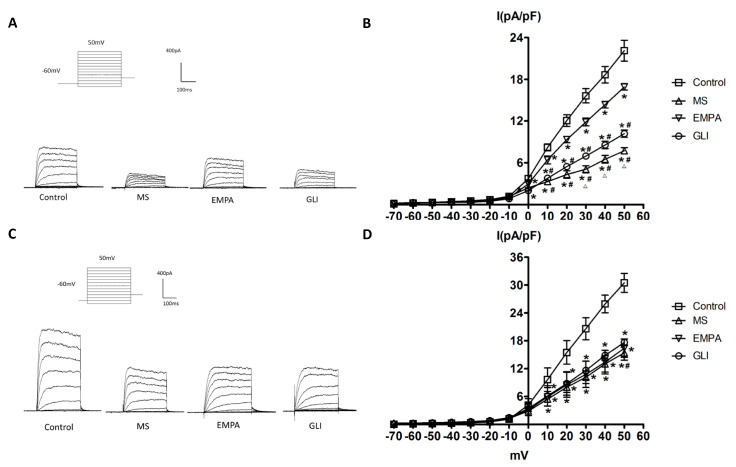
Effects of adipocytokines from the pericardial and peripheral fat tissues of the study groups on the delayed-rectifier potassium current (I_K_) in H9c2 cells. (**A**) Representative recordings of I_K_ elicited by 300 ms of depolarizing step pulses from −70 to 50 mV at a holding potential of −60 mV in H9c2 cells treated with adipocytokines from the pericardial fat of different groups for 18 h. (**B**) Average relationship between the I_K_ (pA/pF) and membrane potential in H9c2 cells treated with adipocytokines from the pericardial fat in the control, metabolic syndrome (MS), empagliflozin (EMPA), and glibenclamide (GLI) groups (*n* = 10). (**C**) Representative recordings of the I_K_ elicited by 300 ms of depolarizing step pulses from −70 to 50 mV at a holding potential of −60 mV in H9c2 cells treated with adipocytokines from the pericardial fat of different groups for 18 h. (**D**) Average relationship between the I_K_ (pA/pF) and membrane potential in H9c2 cells treated with adipocytokines from the peripheral fat in the control, MS, EMPA, and GLI groups (*n* = 10). * *p* < 0.05 compared with the control; # *p* < 0.05 compared with the EMPA; △*p* < 0.05 compared with the GLI. MS: metabolic syndrome; EMPA: empagliflozin; GLI: glibenclamide.

The I_K_ was evoked with 300 ms of depolarizing step pulses from −70 to 50 mV with 10 mV increments at a holding potential of −60 mV in H9c2 cells treated with adipocytokines from the pericardial fat and peripheral fat of the study groups. Figure 2A shows representative current traces of the I_K_ measured in H9c2 cells treated with adipocytokines from the pericardial fat tissue of the study groups. The amplitude of I_K_ increased more when the membrane potentials became more positive in all groups. The amplitudes of I_K_ in the MS, EMPA, and GLI groups were significantly smaller than those in the control group. The average relationships between the I_K_ (pA/pF) currents and the membrane potentials were calculated and are demonstrated in Figure 2B. As shown in Figure 2B, the I_K_ was significantly lower in the MS, EMPA, and GLI groups from 0 to 50 mV than in the control group (*n* = 10, all *p* < 0.05). Compared with that in the EMPA group, the I_K_ was significantly lower in the GLI and MS groups from 0 to 50 mV (all *p* < 0.05). The I_K_ currents in the MS group were significantly lower than those in the GLI group from 30 to 50 mV (all *p* < 0.05).

Figure 2C,D demonstrates the effects of adipocytokines from the peripheral fat tissue of study groups on the I_K_ in H9c2 cells. As Figure 2C demonstrated, the amplitude of I_K_ increased more when the membrane potentials became more positive in all groups. The average relationships between the I_K_ (pA/pF) and membrane potentials were calculated and are shown in Figure 2D. Compared with that in the control group, the I_K_ was significantly lower in the MS, EMPA, and GLI groups from 10 to 50 mV (all *p* < 0.05). However, the I_K_ was not significantly different among the MS, EMPA, and GLI groups (all *p* > 0.05).

### 2.4. The Effects of Adipocytokines on I_Ca,L_ in H9c2 Cells

**Figure 3:**
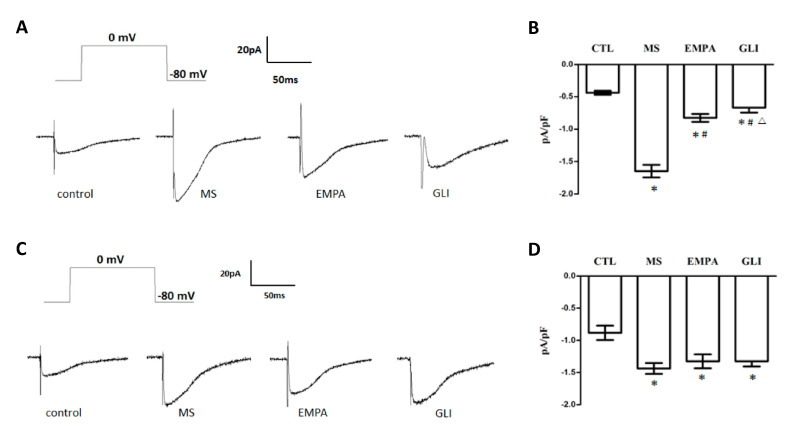
Effects of adipocytokines from the pericardial and peripheral fat tissues of study groups on L-type calcium channel current (I_Ca,L_) in H9c2 cells. (**A**) Representative recordings of the I_Ca,L_ evoked by applying a pulse of 0 mV for 300 ms at a holding potential of −80 mV in H9c2 cells treated with adipocytokines from the pericardial fat of different groups for 18 h. (**B**) Bar graph showing the I_Ca,L_ (pA/pF) averaged from the recordings of the trace (*n* = 6). (**C**) Representative recordings of the I_Ca,L_ evoked by applying a pulse of 0 mV for 300 ms at a holding potential of −80 mV in H9c2 cells treated with adipocytokines from the peripheral fat of different groups for 18 h. (**D**) Bar graph showing the I_Ca,L_ (pA/pF) averaged from the recordings of the trace (*n* = 6) (**C**). Data are the means ± standard deviation. * *p* < 0.05 compared with the control. # *p* < 0.05 compared with the MS; △*p* < 0.05 compared with the EMPA; MS: metabolic syndrome; EMPA: empagliflozin; GLI: glibenclamide.

The perforated whole-cell patch-clump technique was used to study the I_Ca,L_ among the control, MS, EMPA, and GLI groups. Figure 3A shows that a representative recording of the I_Ca,L_ was evoked by a depolarizing single pulse with 0 mV applied for 300 ms at a holding potential of −80 mV in H9c2 cells treated with adipocytokines from the pericardial fat tissue of the study groups. Figure 3B shows the comparison of I_Ca,L_ in H9c2 cells treated with adipocytokines from the pericardial fat of the study groups. The mean I_Ca,L_ of the control, MS, EMPA, and GLI groups were −0.44 ± 0.03, −1.65 ± 0.10, −0.83 ± 0.06, and −0.67 ± 0.08 pA/pF, respectively (*n* = 6). Compared with that in the control group, the I_Ca,L_ was significantly increased overload in the MS, EMPA, and GLI groups (all *p* < 0.01). Compared with that in the MS group, the I_Ca,L_ overload was significantly suppressed in the EMPA and GLI groups (all *p* < 0.01) and the suppressed I_Ca,L_ overload strength in the EMPA group was significantly lower than that in the GLI group (*p* = 0.003) (Figure 3B).

Figure 3C shows that a representative recording of I_Ca,L_ was evoked by a single depolarizing single pulse with 0 mV for 300 ms at a holding potential of −80 mV in H9c2 cells after treatment with adipocytokines from peripheral fat tissue of the study groups, respectively. The mean I_Ca,L_ of the control, MS, EMPA, and GLI groups were −0.88 ± 0.11, −1.44 ± 0.08, −1.32 ± 0.11, and −1.32 ± 0.08 pA/pF, respectively (*n* = 6). Compared with the control group, the I_Ca,L_ overload was significantly increased in the MS, EMPA, and GLI groups (all *p* < 0.01). However, the I_Ca,L_ was not significantly different among the MS, EMPA, and GLI groups (Figure 3D).

## 3. Discussion

In this study, we demonstrated that adipocytokines from the peripheral and pericardial fat tissues of mice with MS could decrease the I_K_ and increase the I_Ca,L_ overload of cardiomyocytes. These effects of decreasing the I_K_ and increasing the I_Ca,L_ overload by adipocytokines from fat tissues in mice with MS could contribute to an increased vulnerability of cardiac arrhythmias. After therapy with EMPA and GLI, the effects of the adipocytokines from pericardial fat tissues on the I_K_ decreasing and I_Ca,L_ overload were attenuated, which might contribute to reducing vulnerability to cardiac arrhythmias in MS with glucose-lowering drug therapy. The attenuation effect of I_K_ decreasing by EMPA was significantly stronger than that by GLI therapy. In contrast, the attenuation of I_Ca,L_ overload by GLI was mildly significantly stronger than that by EMPA therapy. However, adipocytokines from the peripheral fat tissues of mice with EMPA and GLI therapy had no significantly different effects on the I_K_ and I_Ca,L_ of cardiomyocytes.

MS is considered a well-known risk factor for cardiac arrhythmias. MS could significantly increase the volume of pericardial fat and visceral fat [4]. An increased pericardial fat volume is associated with an increased incidence of cardiac arrhythmias [5,6]. Increased adiposis and fibrosis area in atrial and ventricular tissues have been found in individuals with MS [16]. This structural remodeling could decrease conduction velocity and increase conduction heterogenicity in heart tissue, which could contribute to arrhythmogenesis [17]. Several adipocytokines derived from fat tissues have been shown to have effects of inflammation and tissue fibrosis processes [16]. Adipocytokines are also associated with the pathogenesis of atherosclerosis and coronary artery disease [18]. In addition to cardiovascular disease, adipocytokines have also been shown to be involved in cardiac arrhythmias. Lee et al. demonstrated that adipocytokines significantly decreased the I_K_ in ventricular myocytes and that the I_K_ was more prominently decreased by adipocytokines from pericardial fat than from peripheral fat tissues [9]. Lin et al. also demonstrated that leptin, one of the adipocytokines from fat tissue, could alter ion currents in the left atrial myocytes [19]. Taken together, previous and the current study results suggested that beyond structural remodeling, electrophysical remodeling by adipocytokines from pericardial fat also could contribute to arrhythmogenesis in MS.

Sulfonylurea, a traditional glucose-lowering drug, was known to inhibit the adenosine triphosphate-sensitive potassium channels to stimulate insulin secretion. Sulfonylurea might also inhibit the I_K_ and cause QT interval prolongation to increase the vulnerability of cardiac arrhythmias [20]. In this study, we found the EMPA, a new glucose-lowering drug, had stronger attenuation for decreasing the I_K_ by adipocytokines than GLI but mild weaker attenuation for increasing the I_Ca, L_ overload. The total attenuation effects on I_K_ decreasing and I_Ca,L_ overload through modifying adipocytokines might contribute to the fact that the SGLT2 inhibitor had more effects of reducing cardiac arrhythmic vulnerability than GLI.

SGLT2 inhibitors were shown to decrease the incidence of sudden cardiac death in patients with cardiovascular disease in a clinical study [10]. Whether SGLT2 inhibitors have antiarrhythmic effects is still uncertain. Several reports have shown that SGLT2 inhibitors could reduce the adipogenesis of visceral, pericardial fat, and obesity [21,22]. The activation of the PPAR-α and PGC1α pathways by SGLT2 inhibitors has been proposed to result in transcriptional reprogramming to reduce adiposity [23]. In this study, SGLT2 inhibitors also attenuated the effects of adipocytokines from pericardial fat but not peripheral fat on I_K_ and I_Ca,L_ in cardiomyocytes. Compared with sulfonylurea, SGLT2 inhibitors could significantly decrease the level of leptin and IL-6 and increase the level of adiponectin and TNF-α [24]. Different contents of adipocytokines from pericardial and peripheral fat tissue with SGLT 2 inhibitor therapy might contribute to those different effects on alterations of ion currents of cardiomyocytes [25]. Animal studies also showed that the fibrotic area in the heart could be reduced by SGLT2 inhibitors [26]. Based on the previous and current study results, SGLT2 inhibitors could reduce cardiac arrhythmogenesis by reducing structural and electrophysiological remodeling in MS.

## 4. Limitations

There are some limitations to this study. First, although MS mice were created by feeding them a high-fat diet, the serum concentrations of EMPA and GLI in the mice were not determined. Whether the therapeutic effects of EMPA and GLI in the groups were achieved is unknown. However, the different presentations of metabolic characteristics among the study groups may result from different drug therapies. Second, the characteristics of adipocytokines among different groups were not analyzed. The adipocytokines should at least include TNF-α, monocyte chemotactic protein 1, IL-6, IL-8, IL-10, resistin, adiponectin, leptin, plasminogen activator inhibitor 1 angiotensinogen, vascular endothelial growth factors, and other proteins not investigated. A complete analysis of all these cytokines characteristics was difficult. The effects of specific cytokines from adipose tissue on ion currents were not analyzed in this study. Third, the effects of EMPA on the I_K_ and I_Ca,L_ in cardiomyocytes through the modulation of adipocytokines from pericardial fat were confirmed in this study. However, the direct antiarrhythmic effects of EMPA were not demonstrated and the real mechanisms of EMPA affecting ion currents through modifying adipocytokines were not studied completely in this study. Further investigations are needed in the future.

## 5. Materials and Methods

### 5.1. Adipose Tissue Preparation

A total of 20 C57BL/6J mice (age: 8 weeks) were divided into four groups: (1) Five were with standard chow for 16 weeks as the control group, (2) 5 were fed a high-fat diet for 16 weeks as the metabolic syndrome (MS) group, (3) 5 were fed a high-fat diet (Research Diets, New Jersey, USA) for 12 weeks and administered empagliflozin 10 mg/kg daily for the following 4 weeks as the empagliflozin (EMPA) group, and (4) 5 were fed a high-fat diet for 12 weeks and administered glibenclamide 0.6 mg/kg daily for the following 4 weeks as the glibenclamide (GLI) group. After 16 weeks of feeding, all mice were sacrificed. Before sacrificing, 10 c.c. of venous blood was collected from the inferior venous cava to determine the sugar, total cholesterol, triglyceride, low-density lipoprotein, and high-density lipoprotein levels. Pericardial fat and fat of the thigh (peripheral fat) were collected for further investigation, respectively. All study mice were from National Laboratory Animal Center of Taiwan. The study protocol was approved by the Institutional Animal Care and Use Committee at Kaohsiung Medical University (KMU-105107).

### 5.2. Collection of Adipocytokines

Adipocytokines were collected from the pericardial fat and peripheral fat among the study groups, respectively. The techniques of adipocytokine collection have been described in detail in a previous report [7]. In brief, adipose tissue samples were taken from the pericardium and thigh of the mice, respectively. The adipose tissues were rinsed with phosphate-buffered saline (PBS), weighed, cut into small pieces, and then transferred into a 12-well plate. Serum-free Dulbecco’s modified Eagle’s medium (DMEM) was added to the wells and incubated with the fat tissue at 37 °C in a CO_2_ incubator with gentle rocking. After 3 hours, the conditioned media were collected and centrifuged at 4 °C for 10 min. The supernatants containing adipocytokines from the pericardial and peripheral fat tissue cultures were stored in aliquots at −70 °C. The concentration of adipocytokines was quantified by a Bradford protein-binding assay [27].

### 5.3. Cell Culture

H9c2 cells (ATCC CLR-1446; Rockville, MD, USA.) derived from rat embryonic myoblasts are commonly used as an in vitro model of cardiomyocyte biology because they show similar ion currents responses to those seen in primary adult and neonatal cardiomyocytes [28]. Cells were plated onto collagen-coated culture dishes and cultured in DMEM supplemented with 10% fetal bovine serum (FBS) in a humidified atmosphere of 5% CO_2_ and 95% air at 37 °C. Cells were used from the 20th passage.

### 5.4. Determination of Delayed-Rectifier Potassium Outward Currents (I_K_) and l-Type Calcium Channel Current (I_Ca,L_) in H9c2 Cells

The delayed-rectifier potassium outward current (I_K_) in H9c2 cells was measured using the whole-cell patch clamp method. The method regarding the detail manipulation has been described in a previous study [9]. In brief, the H9c2 cells were treated with medium containing adipocytokines (25 μg/mL) derived from central fat and peripheral fat tissues of different groups for 18 h, respectively. After treatment with adipocytokines, the H9c2 cells were detached with 0.25% trypsin-0.02% EDTA solution, and then the supernatant was removed by centrifugation. The pellets were resuspended in 1 mL of bath solution containing 60 mM NaCl, 80 mM Na-gluconate, 0.1 mM CaCl_2,_ 1 mM MgCl_2_, 5 mM KCl, 10 mM HEPES, and 10 mM glucose (pH 7.4, NaOH). A recording electrode was pulled from borosilicate glass (resistance: 4–7 MΩ), and the pipette coated with sticky wax was placed close to the tip to reduce capacitance; it was backfilled with pipette solution containing 0.5 mM MgCl_2_, 30 mM KCl, 110 mM K-gluconate, 10 mM EGTA, 5 mM HEPES, 5 mM Na_2_ATP, and 1 mM GTP-tris (pH 7.2, KOH). The recording electrode and the pipette were gently lowered onto an H9c2 cell to record the ion currents. Negative pressure was briefly applied to rupture the membrane, and a gigaohm seal was obtained. Cells were subsequently voltage clamped. The I_K_ was recorded using an Axopatch 700 A amplifier (Axon Instruments, Union City, CA, USA), filtered at 1 kHz using a low-pass Bessel filter, digitized at 5 kHz, and stored on a computer for subsequent analysis with Clampfit 10.2 (Molecular Devices, San Jose, CA, USA). A 1 M NaCl-agar salt bridge between the bath and the Ag-AgCl reference electrode was used to minimize offset potentials. All electrical recordings were performed at room temperature.

A perforated whole-cell patch clamp technique was used to measure the L-type calcium channel current (I_Ca,L_) in H9c2 cells. The technique was previously described in detail in a previous study [29]. In brief, the H9c2 cells were treated with medium containing adipocytokines (25 μg/mL) derived from central and peripheral fat tissues of different groups for 18 hours, respectively. H9c2 cells were placed in a recording dish and perfused with a bath solution containing 135 mM tetraethylammonium (TEA)-Cl, 1.8 mM CaCl_2_, 2 mM MgCl_2_, 10 mM glucose, and 10 mM HEPES (pH 7.4, Tris). To minimize the outward potassium current, the potassium ions (K^+^) were replaced by cesium ions (Cs^+^) in the pipette solution. A recording electrode was pulled from borosilicate glass (resistance: 3–5 MΩ), and the pipette coated with sticky wax was placed close to the tip to reduce capacitance; it was backfilled with pipette solution containing 140 mM CsCl, 1 mM EGTA, 1 mM MgCl_2_, 5 mM Na_2_ATP, and 5 mM HEPES (pH 7.2, Tris). Membrane currents were recorded using the MultiClamp 700 A amplifier, filtered at 1 kHz using a low-pass Bessel filter, digitized at 5 kHz, and stored on a computer for subsequent analysis with Clampfit 10.2. A 1 M NaCl-agar salt bridge between the bath and the Ag-AgCl reference electrode was used to minimize offset potentials. All electrical recordings were performed at room temperature.

### 5.5. Statistical Analysis

All data are expressed as the mean ± standard deviation. A repeated-measures ANOVA was used to compare differences in data among groups. Continuous variables in the two groups were compared using the nonparametric independent two-sample test (Mann–Whitney U test). A *p*-value <0.05 was considered significant. All statistical analyses were performed using SPSS software 17.0 (SPSS Inc., Chicago, IL, USA).

## 6. Conclusions

Adipocytokines from the pericardial fat of MS mice could decrease the I_K_ and increase the I_Ca,L_ overload of cardiomyocytes. Adipocytokines from pericardial fat but not peripheral fat tissues after SGLT2 inhibitor therapy could attenuate those effects on the I_K_ and I_Ca,L_ of cardiomyocytes, which may contribute to antiarrhythmic mechanisms of SGLT2 inhibitors.

## Figures and Tables

**Table 1:** Characteristics of the study groups at the time of collecting adipose tissues.

	Control	MS	EMPA	GLI
Weight (g)	27.17 ± 1.02	42.61 ± 2.27 *	38.12 ± 4.50 *	38.88 ± 5.21 *
Fasting glucose (mg/dL)	144.60 ± 21.98	155.00 ± 11.86	130.50 ± 20.51	180.40 ± 17.70 *^,#,†^
Cholesterol (mg/dL)	85.28 ± 6.08	133.69 ± 9.04 *	98.92 ± 13.87 ^#^	110.93 ± 4.89 *^,#^
LDL (mg/dL)	9.50 ± 2.03	16.72 ± 2.86 *	11.36 ± 1.56	15.54 ± 1.37 *^,†^
HDL (mg/dL)	75.35 ± 7.13	106.21 ± 9.95 *	94.05 ± 0.73	112.80 ± 5.61 *^,†^
Triglyceride (mg/dL)	64.77 ± 5.35	82.44 ± 15.95	44.41 ± 18.64	36.19 ± 8.91 *^,#^

EMPA: Metabolic syndrome with empagliflozin; GLI: Metabolic syndrome with glibenclamide; HDL: High-density lipoprotein; LDL: Low-density lipoprotein; MS: Metabolic syndrome; *: *p-*value < 0.05 compared with the control group; #: *p-*value < 0.05 compared with the MS group; †: *p-*value < 0.05 compared with the EMPA group.
